# P-56. Clinical and Microbiological Profile of Enterococcal Bacteremia in a Tertiary Hospital in the Dominican Republic

**DOI:** 10.1093/ofid/ofaf695.285

**Published:** 2026-01-11

**Authors:** Rita A Rojas-Fermín, Alfredo J Mena Lora, Anel E Guzmán-Marte, Cristian Adonis Ramon-Santana, Maria Virginia Rodriguez - Pena

**Affiliations:** Hospital General De La Plaza De La Salud, Distrito Nacional, Distrito Nacional, Dominican Republic; University of Illinois Chicago, Chicago, Illinois; Hospital General De La Plaza De La Salud, Distrito Nacional, Distrito Nacional, Dominican Republic; Universidad Nacional Pedro Henriquez Ureña, santo domingo, Distrito Nacional, Dominican Republic; Universidad Nacional Pedro Henriquez Ureña, santo domingo, Distrito Nacional, Dominican Republic

## Abstract

**Background:**

Enterococcal bacteremia is an emerging concern in hospitalized patients, often associated with multidrug resistance and high morbidity. Limited data exists in the Dominican Republic on its clinical features and outcomes.

Antimicrobial resistance patterns by Enterococcus Species

**Figure:**
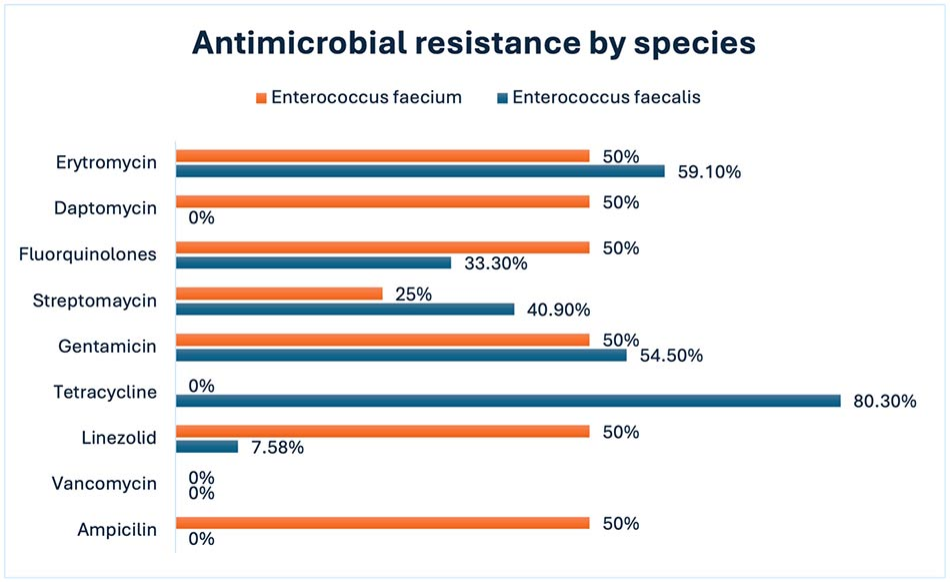

Types of Enterococcus

**Figure:**
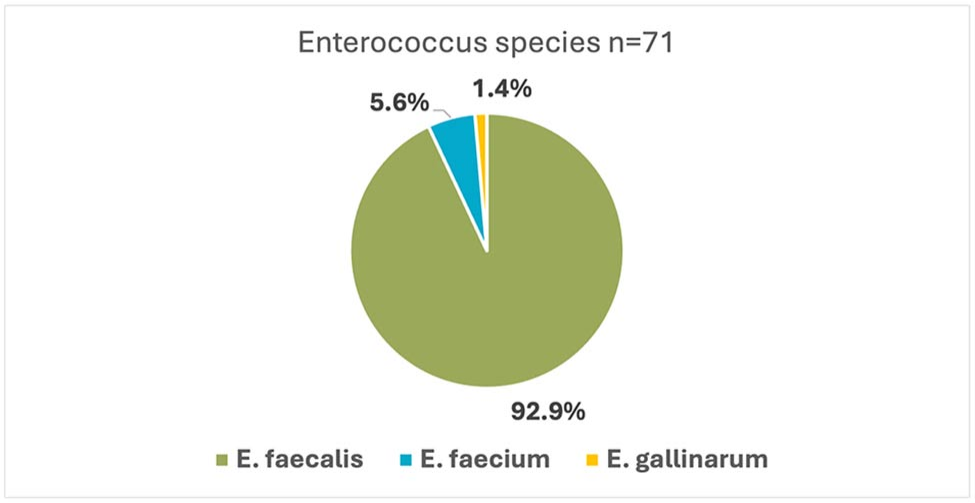

**Methods:**

A retrospective cross-sectional study was conducted at Hospital General Plaza de la Salud from January 2022 to December 2023. Medical records were reviewed to collect demographic, clinical, microbiological, and outcome data on patients with blood culture–confirmed Enterococcus spp. bacteremia.

**Results:**

Seventy-one patients were included, predominantly male (64.7%), with a median age distribution favoring patients over 60 years (64.7%). Comorbidities included chronic kidney disease (43.6%), diabetes (36.6%), and hypertension (18%). Most cases (70.4%) were nosocomial. E faecalis accounted for 92.9% of isolates, followed by E. faecium (5.6%) and E. gallinarum (1.4%). High resistance rates were noted for tetracycline (80.3%), erythromycin (59.1%), and gentamicin (54.5%) among E. faecalis isolates, while 50% of E. faecium isolates showed resistance to ampicillin, linezolid, and daptomycin (Figure 1). No vancomycin resistance was detected.

Vancomycin and ampicillin were the most commonly used treatments, with 70.8% achieving negative blood cultures within 7 days. Bacteremia recurrence occurred in 11.1% of cases. Complications included sepsis (28.1%), endocarditis (8.4%), and pneumonia (7.0%). The overall mortality rate was 28.8%, and 54.1% of patients had hospital stays exceeding 20 days.

**Conclusion:**

Enterococcal bacteremia predominantly affects elderly, vulnerable patients with significant comorbidities in our cohort. E. faecalis remains the most common pathogen, and despite notable resistance patterns, vancomycin and ampicillin retain high efficacy. Early identification, appropriate antibiotic stewardship, and device management strategies are crucial to improve outcomes and prevent recurrences.

**Disclosures:**

Rita A. Rojas-Fermín, MD, GSK: Honoraria

